# Tropical Anemia: One of Africa's Great Killers and a Rationale for Linking Malaria and Neglected Tropical Disease Control to Achieve a Common Goal

**DOI:** 10.1371/journal.pntd.0000270

**Published:** 2008-07-30

**Authors:** Peter J. Hotez, David H. Molyneux

**Affiliations:** 1 Department of Microbiology, Immunology, & Tropical Medicine, The George Washington University and Sabin Vaccine Institute, Washington, D. C., United States of America; 2 Liverpool School of Tropical Medicine, Liverpool, United Kingdom

*“Wiping out malaria would join the eradication of smallpox as one of the greatest accomplishments in human history. It is a goal we can achieve.”*

*Melinda Gates, co-founder,*

*Bill & Melinda Gates Foundation*

With more than 1 million child deaths annually, malaria remains the single leading killer of young children in sub-Saharan Africa [1]. Millions more young children survive, but still suffer from severe anemia and permanent neurological damage [1], as well as more subtle neuropsychiatric disturbances including impaired cognition and memory [2]. Malaria in pregnancy is also a major cause of maternal deaths and low birth weight [3], and together these maternal and child health effects account for huge economic losses that trap families in poverty [4]. As a result, malaria is now considered one of the key forces preventing the development of the African continent [4]. In response to a growing malaria crisis, the Bill & Melinda Gates Foundation recently announced an ambitious program of expanded malaria control, with a long-term goal of malaria eradication [5]. The major elements of expanded malaria control include strengthening of prevention and treatment programs worldwide through the Global Fund to Fight AIDS, Tuberculosis and Malaria, the United States President's Malaria Initiative, the World Bank Malaria Control Booster Program, scale-up of national control programs [5], coordination through the Roll Back Malaria Partnership based at the World Health Organization (WHO) [6], and advocacy by Malaria No More and other organizations [7].

In the early 1970s, an intensified effort to interrupt the transmission of malaria was conducted in a group of villages near the town of Garki in northern Nigeria [8]. Through household spraying, mass drug administration, and other measures, there was a temporary reduction in malaria deaths, but overall the Garki Project showed that interrupting malaria transmission was not possible even when a full armamentarium of control tools was applied [8]. An important difference between then and now is the availability of long-lasting insecticide-treated nets (LLITNs) and artemisinin combination therapy (ACT)-based treatments, in addition to the increased willingness to deploy indoor insecticide spraying [1]. However, it is unclear whether even the deployment of these new control tools will directly lead to total success in malaria control because of the threat of emerging insecticide resistance to pyrethroids and the potential for emergence of artemisinin resistance [1],[9],[10]. Also, parallel efforts will be required to strengthen Africa's weakened health systems [11],[12], which today suffer from widespread malaria misdiagnoses in endemic areas [13] and a lack of access to essential medicines and LLITNs [14]. Accordingly, WHO and other organizations are embarking on renewed efforts to strengthen health systems in Africa and elsewhere [15], while product development partnerships have evolved in a concerted push to accelerate the development of additional new malaria drugs and insecticides, and safe and effective anti-malaria vaccines [1],[5],[16].

**Figure 1:**
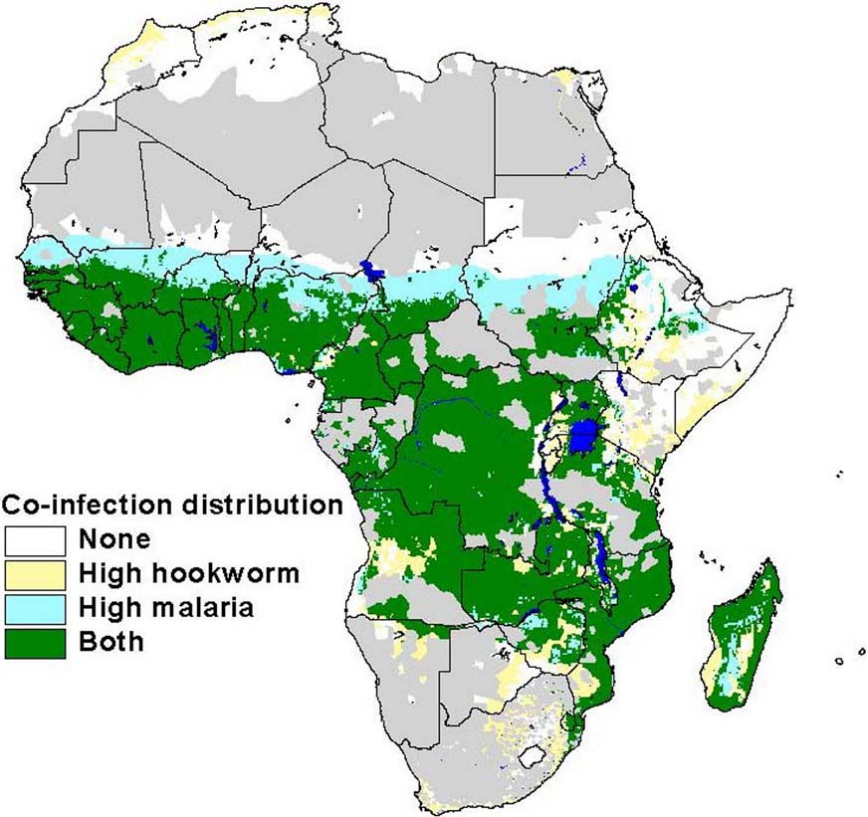
Distribution of Hookworm and Malaria Coinfection. Geographic overlap of moderate-high hookworm infection prevalence (greater than 20% prevalence of infection among school-aged children) and transmission of falciparum malaria transmission (based on a map of climactic suitability for *Plasmodium falciparum* malaria transmission, adjusted for urbanization). Modified from [22].

There is yet another promising, low-cost and highly cost-effective, and complementary approach for potentially reducing the morbidity of malaria in sub-Saharan Africa, which builds on existing efforts and could be implemented for as little as US$0.50 per person per year or less than 10% add-on to projected malaria control costs [17]–[20]. In sub-Saharan Africa, where more than 90% of malaria deaths occur, children and pregnant women are simultaneously infected with both malaria and a group of other parasitic diseases, known as the neglected tropical diseases (NTDs). The major NTDs in sub-Saharan Africa include hookworm infection (198 million cases) and other soil-transmitted helminth infections such as ascariasis and trichuriasis (173 million and 162 million cases, respectively), schistosomiasis (166 million), trachoma (33 million), lymphatic filariasis (46 million), and onchocerciasis (18–37 million) [17],[18]. There is evidence that some of these NTDs exert an adverse influence on the clinical outcome of malaria in childhood and in pregnancy [21]–[24], and even possibly on malaria transmission [25]. Shown in Figure 1 is a previously published map demonstrating the geographic overlap and co-endemicity of falciparum malaria and hookworm infection (Africa's most common NTD), based on statistical and spatial analyses [22]. This analysis shows high spatial congruence of these two infections, with one quarter of all sub-Saharan African schoolchildren simultaneously at risk for hookworm and malaria. Almost all of the estimated 50 million schoolchildren in sub-Saharan Africa with hookworm infection are also at high risk for malaria, except in a small band of the Sahel where the climate is presumably too dry to support the larval development of hookworms [22]. A similar association has also been noted between malaria and schistosomiasis [21]. Therefore, early evidence points to high rates of malaria and NTD coinfections in sub-Saharan Africa, especially with hookworm infection and schistosomiasis.

In Africa and other tropical developing countries, the great killer and disabler that results from malaria and NTD coinfections is anemia [23], [26]–[28]. Anemia accounts for up to one half of malaria deaths in young children [26], and is a leading contributor to the huge numbers of maternal deaths that result during pregnancy, as well as premature births [27]. Chronic anemia in young children is also associated with reductions in physical growth, and impaired cognition and school performance [23],[24]. Many of the NTDs, but especially two of the most common ones in sub-Saharan Africa, hookworm infection and schistosomiasis, cause anemia [24], [28]–[32], while in Asia (and presumably elsewhere), hookworm infection, schistosomiasis, and trichuriasis result in a synergistic anemia [33]. Malaria also causes severe anemia [26],[27], and in cases of malaria and NTD coinfection, anemia in vulnerable children and women develops through one or more of several mechanisms including blood loss, hemolysis, anemia of inflammation, and splenic sequestration [22]–[24], [28]–[32]. An important consequence of malaria and NTD coinfections is an enhancement in anemia, or what we have called previously “the perfect storm of anemia” [24]. For instance, in Kenya, hemoglobin concentrations were found to be 4.2 g/l lower among children harboring hookworm and malaria coinfections than in children with single-species infections [23]. Because hookworm and schistosomiasis are widespread in Africa [17],[21],[22], it is likely that these NTDs represent important contributors to the overall mortality from childhood malaria in this region [23],[24],[28].

Similarly, most of the 7.5 million pregnant women infected with hookworm likely live in areas of sub-Saharan Africa that place them at risk for malaria [27],[31]. At the same time, malaria control and NTD control have each been shown to reduce anemia both in children [23],[26],[34],[35] and in pregnant women [30],[31],[36],[37]. Therefore, combining malaria and NTD control practices in a unified anemia framework affords one of the best opportunities to reduce the huge burden of morbidity and mortality that results from anemia in sub-Saharan Africa. In addition to the health improvement that would result from anemia reduction, there is also some evidence that hookworm and schistosomiasis (and possibly other NTDs) may immunomodulate their human host and promote increased susceptibility to malaria, so that NTD control would work in synergy with nets and other measures to reduce malaria incidence [25].

In sub-Saharan Africa, there are several opportunities to link malaria and NTD control programs [23]. They include programs targeted for infants, preschool children, or school-aged children that employ intermittent preventive treatment (IPT), in which use of either sulfadoxine–pyrimethamine or ACT [38] would be supplemented with anthelminthic drugs (“deworming”) or with a rapid-impact package of NTD drugs that simultaneously target hookworm and other soil-transmitted helminth infections, lymphatic filariasis, onchocerciasis, and trachoma [17],[23],[24]. A joint program of malaria and NTD control could be incorporated as a new element of Integrated Management of Childhood Illness and other programs for children [39]. There are also opportunities for linking IPT in pregnancy with anthelminthic drugs (or the rapid-impact package) for NTD control, especially given the benefit of deworming in terms of improving birth outcome and reducing maternal morbidity and mortality [36],[37]. The anthelminthic drugs mebendazole and albendazole can be used in the second and third trimester of pregnancy and are recommended by WHO in the appropriate settings [40]. Community-based prevention efforts could also be integrated. Both untreated bed-nets and LLITNs are proportionately more effective in preventing lymphatic filariasis compared with malaria [41], and the use of bed-nets was shown to increase substantially, in some cases 9-fold, when used alongside NTD control efforts [42].

Together, malaria and the seven most common NTDs listed above cause almost 2 million deaths and are responsible for the loss of almost 100 million disability-adjusted life years (DALYs) annually (almost 20% higher than the disease burden from HIV/AIDS) [23]. Much of this high disease burden operates through the mechanism of anemia. According to J. Crawley, “…an integrated and non-disease specific approach is essential if the intolerable burden of anemia that currently exists in malaria-endemic regions of Africa is going to be reduced” [43]. Although there will be operational challenges to integrating NTD with malaria control, the opportunities for improving health, education, and economic development for the poorest people in sub-Saharan Africa are simply too great for us to ignore. Accordingly, the public–private partnerships of the Global Network for NTDs are working to identify opportunities for integration [18]. In addition, given the ability of hookworm and other NTDs to interfere with vaccine immunogenicity, we also need to consider the importance of NTD research for the malaria research agenda [44], as well as the opportunity to develop new NTD drugs and vaccines alongside new innovations in malaria treatment and prevention [45].

It is likely that focusing control efforts on malaria alone will thwart global efforts to sustain malaria control, much less achieve eradication. Ultimately, by reducing anemia in sub-Saharan Africa, linking the NTDs with malaria control would have a major impact on almost all of the Millennium Development Goals [20]. It is some four years since this approach was suggested [19], but policy makers are only gradually recognizing the benefits of more holistic approaches to tackling the diseases of the poor. An integrated control program for tropical anemia in Africa represents one of our better hopes for a quick win in the fight for sustainable disease control and poverty reduction.
